# Hydrothermally Grown In-doped ZnO Nanorods on p-GaN Films for Color-tunable Heterojunction Light-emitting-diodes

**DOI:** 10.1038/srep10410

**Published:** 2015-05-19

**Authors:** Geun Chul Park, Soo Min Hwang, Seung Muk Lee, Jun Hyuk Choi, Keun Man Song, Hyun You Kim, Hyun-Suk Kim, Sung-Jin Eum, Seung-Boo Jung, Jun Hyung Lim, Jinho Joo

**Affiliations:** 1 School of Advanced Materials Science and Engineering, Sungkyunkwan University, Suwon, Gyeonggi 440-746, Korea; 2 Department of Nanomaterials Engineering, Chungnam National University, Daejeon 305-764, Korea; 3 Department of Materials Science and Engineering, Chungnam National University, Daejeon 305-764, Korea; 4 HeeSung Material Ltd. 113-9, Yongin, Gyeonggi, 449-884, Korea

## Abstract

The incorporation of doping elements in ZnO nanostructures plays an important role in adjusting the optical and electrical properties in optoelectronic devices. In the present study, we fabricated 1-D ZnO nanorods (NRs) doped with different In contents (0% ~ 5%) on p-GaN films using a facile hydrothermal method, and investigated the effect of the In doping on the morphology and electronic structure of the NRs and the electrical and optical performances of the n-ZnO NRs/p-GaN heterojunction light emitting diodes (LEDs). As the In content increased, the size (diameter and length) of the NRs increased, and the electrical performance of the LEDs improved. From the electroluminescence (EL) spectra, it was found that the broad green-yellow-orange emission band significantly increased with increasing In content due to the increased defect states (oxygen vacancies) in the ZnO NRs, and consequently, the superposition of the emission bands centered at 415 nm and 570 nm led to the generation of white-light. These results suggest that In doping is an effective way to tailor the morphology and the optical, electronic, and electrical properties of ZnO NRs, as well as the EL emission property of heterojunction LEDs.

ZnO, which possesses a wide band-gap (3.37 eV) and a high exciton-binding energy (60 meV), is a promising n-type semiconducting material in optoelectronic devices such as solar cells, laser diodes (LDs), and light emitting diodes (LEDs)^1^,^2^,^3^,^4^. The 1D nanostructures based on this material, especially nanowires (NWs) and nanorods (NRs), have recently attracted considerable attention for practical application in LEDs because they provide effective conduction paths for electrons due to their high crystallinity compared to a thin-film structure^5^,^6^,^7^. Vertically-aligned ZnO NWs/NRs can also act as direct waveguides that minimize the side scattering of the light, and thus enhance the light extraction efficiency without use of reflectors and lenses^8^. Despite the superior properties of the 1D ZnO nanostructures and the remarkable advance in the synthesis methods towards high-quality ZnO, the successful implementation of nanostructure-based LEDs remains challenging because of the difficulties in assembling high-performance integrated devices as well as in constructing stable p-n homojunctions due to their intrinsic n-type nature^9^,^10^. In an effort to circumvent this issue, substantial researches have been devoted to fabricate heterojunction LEDs by growing n-type ZnO NRs on other p-type materials^11^,^12^,^13^. p-GaN is commonly used as a suitable candidate as it has the same lattice structure and similar band gap energy as those of ZnO^14^.

However, the emission efficiency and output power of the n-ZnO NRs/p-GaN heterojunction LEDs are insufficient to achieve high performance devices due to their lower carrier injection efficiency, resulting from a high energy barrier at the junction interface (type-II band alignment: staggered heterojunction)^15^. This problem can be overcome by the incorporation of foreign elements in the ZnO NRs such as Al, Ga, In, Cl, Sn, Cu, and Br, etc., which can change the crystallinity, geometry (including size and morphology) and electronic property of the NRs, thereby enhancing the electrical and optical properties of the LEDs. The incorporated doping elements also enable the tuning of the light emissions of the LEDs. Studies on color-tuning have recently become prominent because of the growing demand for white-LEDs which have many advantages over traditional single light LEDs, such as lower energy consumption and higher transfer efficiency^16^,^17^,^18^. Through the doping process, we expect that the white-light LEDs can be achieved without using phosphors or mixing several colored lights being used in conventional LEDs.

Among various n-type dopants such as Al, Ga, In, Cl, and Br, In is recognized as one of the most efficient elements to satisfy both the requirements of enhancing the LED performances and tuning the emission colors. Moreover, In-doped ZnO NRs has excellent optical transmission, high electrical conductivity, and chemical stability^19^,^20^,^21^. For these reasons, many studies have been conducted to incorporate In into ZnO NRs using various synthesis methods, and In-doped ZnO NRs have been applied to practical applications of optoelectronic devices^22^,^23^,^24^,^25^,^26^. However, obtaining high quality crystallinity with enhanced optical and electrical properties for the realization of high efficiency devices remains a challenge. Furthermore, the correlation between defect states and electrical/optical properties has not been systematically studied.

In this study, we fabricated n-ZnO NRs/p-GaN heterojunctions using a facile hydrothermal method, in which In was doped into the ZnO NRs to improve the electrical conductivity and to tune the optical band-gap energy, and the LED performances were examined by varying the In content. To the best of our knowledge, the application of the In-doped ZnO NRs to the heterojunction LEDs has not yet been reported elsewhere. It was found that the growth behavior, optical energy band gap, and electronic property of the ZnO NRs, and the electroluminescence (EL) of the LEDs varied with the different In doping contents. This work provides an effective path for developing nanostructure-based devices for color-tunable LEDs.

## Results

**Figure 1:**
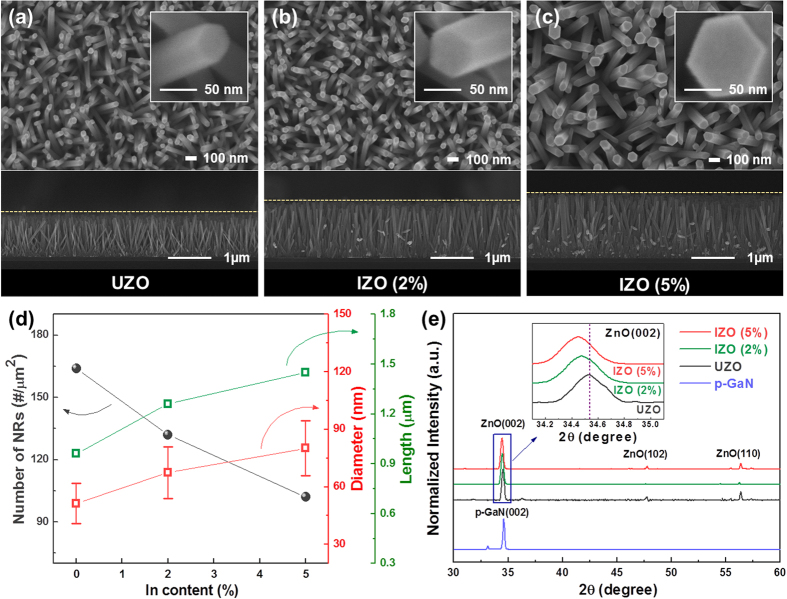
Top-view and cross-sectional FE-SEM images of the IZO NRs with different In concentrations: (**a**) 0%, (**b**) 2%, and (**c**) 5%, and (**d**) variations of the average diameter, length, and density. (**e**) XRD patterns of the p-GaN film and UZO and IZO (2% and 5%) NRs. The inset in Fig. 1(e) shows the enlarged view of the (002) peak.

Figure 1a-c and their insets show the top-view and cross-sectional FE-SEM images for the morphologies of the UZO and IZO NRs on the p-GaN/Al_2_O_3_. For all the samples, the hexagonal-shaped NRs were vertically grown on the p-GaN film, and the surface of the NRs was clean and no notable impurities were present. The average diameter, length, and the number of NRs per unit area (density), which were estimated from the corresponding SEM images, are plotted in Fig. 1d. The values of the average diameter, length, and density of the UZO sample were approximately 51.28 ± 10.53 nm, 0.96 ± 0.03 μm, and 164 rods/μm^2^, respectively. As the In content increased, the average diameter and length increased, while the density decreased. Figure 1e exhibits the XRD patterns of the p-GaN film and UZO and IZO NRs. Both the p-GaN film and ZnO NRs peaks corresponded to a wurtzite hexagonal structure, and any secondary phases such as In_2_O_3_ and In(OH)_3_ were not found for the IZO samples. The strong and dominant (002) peak for the ZnO samples indicates that the NRs grew along the direction perpendicular to the p-GaN film. As can be seen in the enlarged view of the (002) peaks (inset of Fig. 1e), the (002) peak of the IZO NRs shifted toward a lower 2θ with increasing the In content, indicating an increase of the c-axis lattice constant. The variation in the lattice constant was probably due to the different ionic radii of In (r(In^3+^) = 0.081 nm) and Zn (r(Zn^2+^) = 0.074 nm). This result demonstrates that the larger In atoms were substituted for the smaller Zn sites in the ZnO lattice.

**Figure 2:**
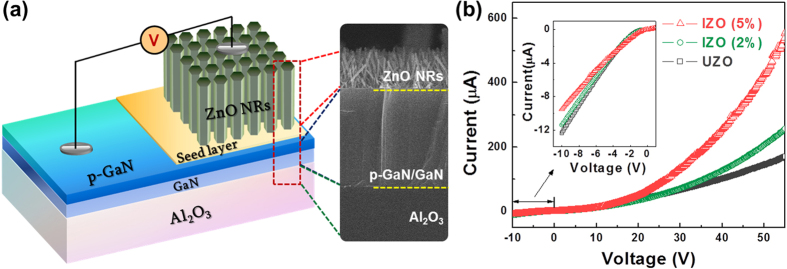
(**a**) Schematic diagram and (**b**) I-V characteristics of the UZO and IZO NRs/p-GaN heterojunction LEDs.

In order to evaluate the effect of In doping on device performances, the electrical and optical properties of the n-ZnO NRs/p-GaN heterostructure LEDs were investigated. A schematic diagram and a cross-sectional FE-SEM image of the n-ZnO NRs/p-GaN heterojunction LED are shown in Fig. 2a. The p-GaN acted not only as a buffer-layer to grow ZnO NRs epitaxially, but also as a p-type material for the hole injection layer in the LEDs. The *I*-*V* curves of the LEDs are shown in Fig. 2b, which show the rectifying behavior with a turn-on voltage of about 5 V. The small breakdown voltage under reverse bias indicates the presence of a leakage current at the junction, and this may be ascribed to not only the contact resistance between the NRs and metal electrode, but also the defect-mediated tunneling effect caused by the high defect concentration or trap centers in the interfacial layer^27^,^28^. From the *I*-*V* curves, it was clearly observed that the current increased with increasing voltage and the increment became more significant as the In content increased: the current of the UZO and IZO (5%) was 142 and 425 μA, respectively, at 50 V. Significant increases of the slope in the *I*-*V* curves for the IZO suggest that the electrical conductivity was enhanced with increasing In content due to the increased charge carriers (electrons) in the IZO NRs. The increased conductivity of NR by In doping is consistent with other studies of ZnO nanostructures such as nanobelts or NWs^29^,^30^. Su *et al.* synthesized In-doped ZnO nanobelts via thermal evaporation and demonstrated that the resistivity of the nanobelts decreased as the In content increased. In addition, it is reported that transparent field-effect transistors (FETs) with In-doped ZnO NWs exhibited a higher mobility than the FETs with un-doped ZnO NWs.

**Figure 3:**
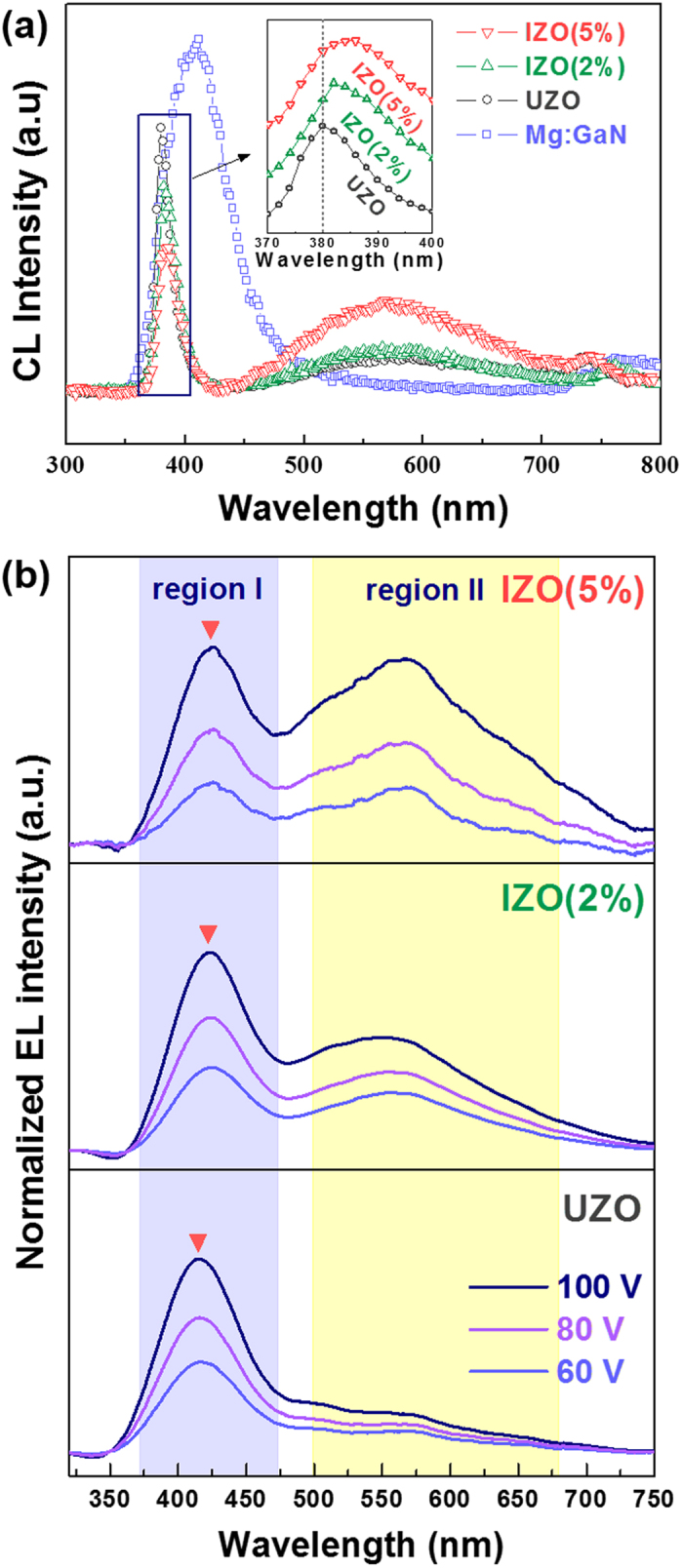
(**a**) CL spectra of the p-GaN film and IZO (0 ~ 5%) NRs. (**b**) EL spectra of the UZO and IZO NRs/p-GaN heterojunction LEDs.

CL measurement was carried out to examine the optical properties of the UZO and IZO NRs, such as energy band gap and defect-related visible emissions. As depicted in Fig. 3a, the emission peak from the p-GaN was red-shifted from the band gap to around 412 nm. This shift is generally observed in Mg-doped GaN films because of the dominant band to acceptor transition^31^. On the other hand, the CL emissions that originated from the ZnO NRs showed two dominant bands. One band was a strong UV emission peak located at about 380 nm, which was attributed to the recombination of free excitons at the near band edge (NBE). The other band was observed as a long tail at about 570 nm, which was associated with the electron-hole recombination at deep levels (DLs) caused by intrinsic defects such as oxygen vacancies and/or zinc interstitials^32^.

The UV peaks of the IZO samples were located in a longer wavelength region compared to the UZO sample. This red-shift of the UV peak can be explained by the anomalous energy shift and band gap renormalization (BGR) effect^33^,^34^,^35^ in CL emission spectra. In the study by Chen *et al*, the UV emission peak of ZnO NRs showed blue-shift as the diameter decreased from 180 to 50 nm. Below a critical diameter (d < 620 nm), a linear relationship between the energy shift (∆E_g_) and inverse of diameter (1/d) was demonstrated from the experimental and calculative data. According to their results, the anomalous energy shift was caused by the surface resonance effect, and the shift became significant as the diameter of NRs decreased. In the same manner, the average diameter of the ZnO NRs in our case increased from 51 to 80 nm with increasing In content (0% ~ 5%), in which the diameter for all the samples was lower than the critical value. Therefore, the red-shift in the present study may be ascribed to the relative decrease of the surface-to-volume ratio, resulting from the increase in the NR size. In addition, the BGR effect probably originated from the many body effects in IZO NRs, which are related to the mutual exchange and coulomb interactions between the added free electrons in the conduction band and electron-impurity scattering. The increased electrons due to the In doping could hinder the free exciton transitions, and this screening could lead to an increase in the energy of the valence band maximum (VBM) and decrease in the energy of the conduction band minimum (CBM). Therefore, the red-shift of the UV peak was probably caused by the combined effects of the increased NR size and the BGR effects by incorporating In.

On the other hand, the relative intensity of the visible emission to the UV emission increased with increasing In content. The increased intensity ratio was due to the increase of the oxygen vacancies by the In doping. This result coincides well with the SEM-panchromatic CL images. As shown in Fig. S1, the UZO sample exhibited a strong and dominant UV emission (blue color), but a very weak or negligible visible emission (yellow color). In contrast, the visible emission in the CL image increased after In doping. Therefore, these results suggest that incorporating the In element enables adjustment of the UV and visible emissions of the ZnO NRs. The variation of the oxygen vacancy concentration with In content will be addressed later with XPS analysis.

Figure 3b presents the normalized EL spectra of the UZO and IZO LEDs operated at bias voltages of 60, 80, and 100 V, and the integrated EL intensity as a function of the applied voltages is plotted in Fig. S2. The EL spectra for all the samples showed two emission bands, one centered at 412 nm (region I) and the other at a broad covering range from 485 to 700 nm (region II). The light emission from the LEDs was observed under the forward bias, while no emission was detected under the reverse bias due to insufficient carrier transport in the n-ZnO/p-GaN heterojunction, which concurs with most n-ZnO/p-GaN LED reports^6^,^11^,^36^,^37^. Under the forward bias, the electrons in the conduction band of ZnO NRs move towards the p-GaN, and the holes in the valance band of p-GaN move towards the ZnO NRs. As the forward bias increased from 60 to 100 V, the EL intensity for all the samples gradually increased without significant change in both the shape and position of the peak. The enhancement in the EL intensity was attributed to the activation of more radiative recombination centers in both the ZnO NRs and p-GaN. This behavior is related to the band bending model of a p-n junction^38^: As voltage increases, the band bending between the p and n materials is reduced, and thus, more carriers are able to traverse on the opposite side of the junction.

By comparing the EL spectra shown in Fig. 3b with the CL spectra shown in Fig. 3a, it can be clearly seen that the EL peak position of the blue-violet emission in region I is close to that of the peak of p-GaN centered at ~412 nm in the CL spectra, and the green-yellow-orange emission band position in the EL spectra (region II) is almost the same as the visible emission peak of the ZnO NRs centered at ~570 nm in the CL spectra. On the other hand, the UV peak located at about 380 nm from the ZnO NRs in the CL spectra was not observed in the EL spectra, which suggested that the radiative recombination is mainly generated from the DL of the ZnO NRs. The origin of the EL emissions can be understood from the energy band diagram model (Fig. S3). As shown in the diagram, the conduction band offset (ΔE_c_ = χ_ZnO_ - χ_GaN_) is 0.15 eV, and the valance band offset (ΔE_v_ = E_g,ZnO_ – E_g,GaN_ + ΔE_c_ ) is 0.13 eV, where the E_g_ values of ZnO and GaN are 3.37 eV and 3.39 eV, respectively. The origin for the emission bands in the regions I and II differs: The emission band in region I most likely resulted from a radiative recombination from the conduction band to the acceptor level induced by Mg doping in the p-GaN rather than from the NBE of ZnO NRs. This is because the electron mobility in the ZnO NRs is higher than that of the holes in p-GaN due to the difference in the effective mass and carrier scattering mechanism. On the other hand, the emission band in region II was probably attributed to the transition from the conduction band to the defect states in the ZnO NRs.

According to the results obtained by other groups^39^,^40^, the EL emissions of the LEDs originated from the various transitions from not only the n-ZnO and p-GaN, but also the p-GaN/ZnO interface. The radiative recombination at the junction interface depends on the magnitude of the applied voltage^11^. When the applied voltage is higher than the threshold value, the barrier at the interface will become thinner, so that the electrons in the conduction band of the ZnO NRs and the holes in the valance band of GaN pass through the interface without recombination. Therefore, the radiative recombination from the junction interface in the present study might rarely be generated since the applied voltages were much higher than the threshold values (V < ~10 V).

It is also noted that the EL emission in regions I and II varied with the In content. With increasing the In content, the EL intensity in both the regions increased, but the peak shift was observed only in region I. The increased intensity and red-shifted peak in region I appeared to be the result of the increased charge carrier injection from the IZO NRs into the p-GaN. At the high concentration of charge carriers in the IZO NRs, the electron-hole recombination in both the p-GaN and IZO NRs increases, which results in an increase of light emission of the LEDs. In addition, the excess carrier injection leads to band gap narrowing in the p-GaN^41^, which in turn causes an increase of nonradiative recombination as well as a red-shift of the emission band^42^. On the other hand, the variations of EL intensity in region II with In doping content was related to the defect states in the IZO NRs. From the energy diagram of IZO NRs/p-GaN (Fig. S3), the electrons in the conduction band will recombine with the defect sites in the IZO NRs and produce a light emission. As the In content increased, the concentration of the oxygen vacancies (related to defect states) increased, resulting in the increase of the EL intensity in the range of 475–700 nm. Consequently, the LEDs emitted the white-light as a result of the superposition of two dominant emission bands near the blue-violet and green-yellow-orange region, and the white-light even able to be observed with even the naked eye.

**Figure 4:**
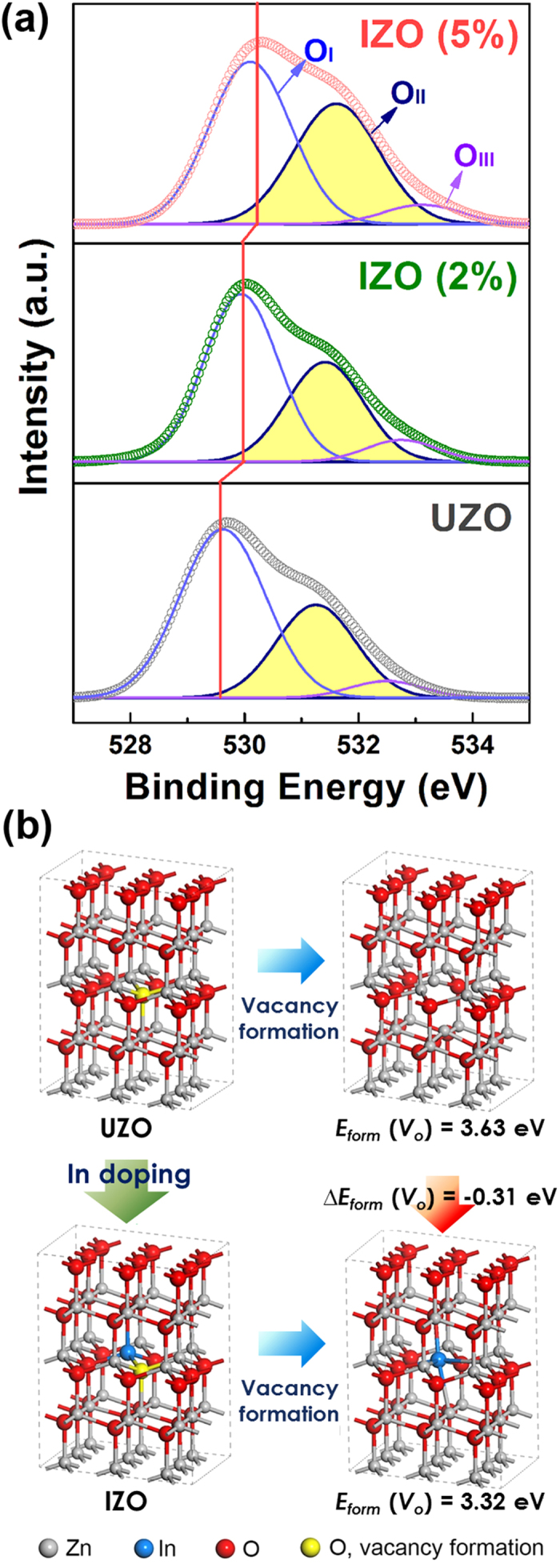
(**a**) XPS spectra of the deconvoluted O 1s of the IZO (0% ~ 5%) NRs. Schematic illustration of the formation mechanism of oxygen vacancy in (**b**) UZO and IZO system.

In order to examine the electronic structure changes, such as chemical bond configurations and defect states, and to understand the CL and EL emission behaviors due to the In-doping, XPS and DFT calculations were performed on the UZO and IZO NRs. Figure 4a shows the narrow-scan O 1s peaks of the samples. The O 1s and Zn 2p peaks (Fig. S4) of the IZO samples shifted slightly toward higher binding energy as the In content increased. Both the values of peak shift for O 1s and Zn 2p spectra are 0.6 eV for the IZO (5%) sample. These peak shifts were attributed to the difference in the electronegativity (χ) of Zn (χ = 1.65) and In (χ = 1.78), which leads to the increases in the binding energy of the Zn 2p and O 1s peaks^20^.

The asymmetric peaks observed in the O 1s were deconvoluted into three types of oxygen levels^43^: the component with low binding energy (O_I_) corresponds to the O^2−^ bonded with metal ions (Zn^2+^ and In^3+^) in the ZnO lattice. The medium binding energy component (O_II_) is attributed to the O^2−^ ions in the oxygen-deficient regions, indicating the concentration of the oxygen vacancies. The high binding energy peak (O_III_) is related to chemisorbed or dissociated oxygen species, such as hydroxyl groups (–OH) and H_2_O, strongly bound to surface defects. Notably, as the In content increased, the intensity ratio of O_II_/O_total_ increased from 32% to 41%, indicating an increase of the oxygen vacancy concentration. This was probably due to the lower bond strength of In to oxygen than that of Zn to O^44^.

The results of the DFT calculations using a ZnO supercell are illustrated in Fig. 4b. The formation energy of an oxygen vacancy *E*_*form*_(*V*_*o*_) was estimated as follows:


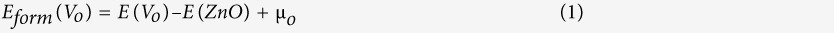


where *E*(*V*_*o*_) represents the energy of a ZnO supercell (with or without In) with a single oxygen vacancy, *E*(ZnO) is the energy of a ZnO perfect crystal, and *μ*_o_ denotes the oxygen chemical potential which is determined by the energy of an O_2_ molecule. The *E*_*form*_(*V*_*o*_) was calculated by removing the 1st nearest oxygen ion from the Zn ion in UZO and the In ion in IZO. The *E*_*form*_(*V*_*o*_) of the IZO (3.32 eV) was lower than that of the UZO (3.63 eV) by 0.31 eV. We postulate, therefore, that the incorporation of In in ZnO NRs would promote the formation of oxygen vacancy defects by lowering the *E*_*form*_(*V*_*o*_). The XPS O 1s spectra and DFT calculations demonstrate that the In doping affects the visible region in both the CL emissions and the EL emissions due to variations in the defect states of the ZnO NRs.

## Discussion

In the present study, we synthesized UZO and IZO NRs on p-GaN films using the hydrothermal method, and investigated the effects of In doping on the morphology, microstructure, chemical bonding state, and optical property of the ZnO NRs and the resulting LED performances were evaluated. As the In content increased, the sizes such as the average diameter and length of the ZnO NRs increased, while the density decreased, which indicates that the growth behavior of the ZnO NRs is strongly affected by incorporating the In element. According to the theoretical and experimental reports on hydrothermally grown In-doped ZnO nanostructures^45^,^46^,^47^, it is expected that In ions were released from the In nitrate hydrates, and incorporated in the ZnO lattice during the growth process, as shown in equations (2)–(6), 3, 4, 5, 6.


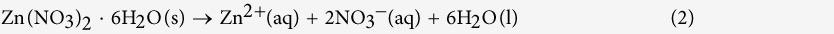



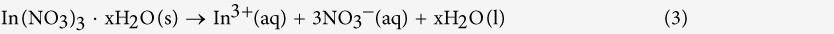



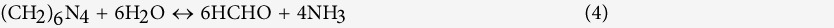



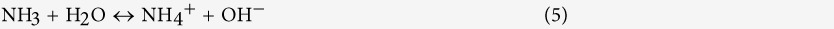



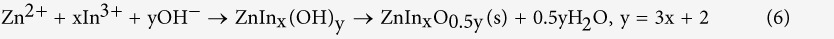


The increased NR size (diameter and length) by In doping can be attributed to the inhibition of the heterogeneous nucleation of the ZnO and the reduced concentration of OH- ions in the growth solution. The growth mechanism of the doped ZnO NRs was described in detail in our previous reports^48^.

From the XRD and XPS results, the increase in the c-axis lattice constant of the IZO NRs and the increases in the binding energy of Zn 2p and O 1s peaks indicated that the In dopants were incorporated in the ZnO NRs. As the In content increased, the UV peaks of the IZO samples shifted slightly toward the longer wavelength because of the combined effects of the increased NR size and the BGR effects. In addition, it was found that the defect states in the ZnO NRs increased due to the lower bond strength of In-O than that of Zn-O, as demonstrated in the XPS O1s and CL spectra. These results suggest that the In doping enables the tuning of the UV and visible emissions of the ZnO NRs.

The incorporated In ions strongly affected the electrical and EL properties of the IZO NRs/p-GaN LEDs. The electrical conductivity and emission efficiency of the LEDs were enhanced due to the increased radiative recombination, resulting from the increased charge carrier concentration in the ZnO NRs. Simultaneously, the EL emission of the n-ZnO NRs/p-GaN heterostructure LEDs varied with the In content. The EL intensity in the range of 475–700 nm increased as the In content increased due to the increased concentration of the defect states (related to oxygen vacancies). The white-light was successfully produced through the superposition of the blue-violet emission from the p-GaN and green-yellow-orange emission from the defect states of the IZO NRs. The EL spectra (Fig. 3b) and band diagram model (Fig. S3) show that the incorporation of In in ZnO NRs allow the color of the LEDs to be tailored and provide an effective way to achieve white-light LEDs by controlling the defect states.

## Methods

### Experimental

In-doped ZnO NRs were synthesized on GaN/Al_2_O_3_ (001) substrates by a low temperature hydrothermal growth process. The Mg-doped GaN films were grown on GaN buffer layer/c-Al_2_O_3_ substrates using metal organic chemical vapor deposition (MOCVD). The thickness and hole concentration of the p-GaN layer were approximately 0.36 μm (pre-deposited GaN buffer layer is 2 μm) and 7.5 × 10^18^ cm^−3^, respectively. To grow ZnO NRs on the p-GaN substrates, in the first step, a ZnO seed layer was deposited on the substrate by spin-coating. A 0.05 M precursor sol was prepared by dissolving zinc acetate dihydrate (Zn(CH_3_COO)_2_⋅2H_2_O) in 2-methoxyethanol, which was then spin-coated at 3000 rpm for 30 s on the p-GaN layers. The seed layer-coated substrates were then immersed in an aqueous solution containing a mixture of 0.025 M zinc nitrate hexahydrate (Zn(NO_3_)_2_∙6H_2_O) and equivalent molar hexamethylenetetramine (HMT, C_6_H_12_N_4_) at approximately 95 ^o^C for 4 h. To obtain In-doped samples, Indium nitrate hydrate (In(NO_3_)_3_∙xH_2_O) was added to the aqueous growth solution with nominal concentrations of 2 and 5 mol%. Hereafter, undoped ZnO and In-doped ZnO are denoted as UZO and IZO, respectively. In order to evaluate the LED performances, In electrodes were deposited onto the ZnO NRs and p-GaN films by thermal evaporation.

### Characterization

The morphology and size distribution of the NRs were observed using field emission scanning electron microscopy (FE-SEM, JEOL, JSM7000F) operated at 15 kV. The crystallographic information was obtained by X-ray diffraction (XRD, Bruker-AXS, D8 Discover) and the chemical bonding structure was analyzed through X-ray photoelectron spectroscopy (XPS, VG Microtech, ECSA2000) using Al *Kα*. A Shirley function was used to subtract the background and all the spectra were fitted by using a Gaussian–Lorentz function, i.e. Gaussian/Lorentz = 80/20. The optical property was measured using cathode-luminescence (CL) spectroscopy (GATAN, MONO CL^3+^) at room temperature. The current-voltage (*I*-*V*) measurement of the n-ZnO NRs/p-GaN heterojunction LEDs was performed with a semiconductor parameter analyzer (Agilent, B1500A) and the EL property of the LEDs was evaluated using voltage/current source meter (Keithley Instruments, Keithley 2400) and UV-visible spectrophotometer (Ocean optics, USB 4000). The EL spectra were smoothed by using Savitzky-Golay algorithm and were finally normalized to the maximum value.

### Simulation

In order to understand the energetics of the oxygen vacancy formation in ZnO NRs by In doping, spin-polarized density-functional theory (DFT) calculations in a plane-wave basis were performed using the Vienna ab-initio simulation package (VASP) code and the Perdew-Burke-Ernzerhof generalized gradient approximation (PBE GGA) functional^49^,^50^. Valence electrons and core electrons were described by plane waves up to an energy cutoff of 500 eV and the projector augmented wave framework, respectively^51^. A 3 × 3 × 2 ZnO (wurtzite structure) supercell with 72 atoms was applied for the calculation of the oxygen vacancy formation energy. We used 3 × 3 × 3 k-points grid sampling of the Brillouin zones for all calculations. The final convergence criteria for the electronic wave function and geometry were 10^−4^ eV and 0.02 eV/Å, respectively.

## Author Contributions

G.C.P., J.H.L. and J.J. suggested the original main idea of this work and G.C.P., S.M.H. and K.M.S. synthesized and fabricated the In-doped ZnO nanorods and LED device. S.M.L., J.H.C., J.H.L., S.E. and S.B.J. performed experiments and analyzed data. H.Y.K. and H. Kim carried out the spin-polarized density-functional theory (DFT) calculations. G.C.P. and J.J. wrote the manuscript. All authors discussed the results and commented on the manuscript.

## Additional Information

**How to cite this article**: Park, G.C. *et al.* Hydrothermally Grown In-doped ZnO Nanorods on p-GaN Films for Color-tunable Heterojunction Light-emitting-diodes. *Sci. Rep.* **5**, 10410; doi: 10.1038/srep10410 (2015).

## Supplementary Material

Supplementary Information
