# Supplementary material for: Hydrothermally Grown In-doped ZnO Nanorods on p-GaN Films for Color-tunable Heterojunction Light-emitting-diodes
Source: Sci Rep. 2015 May 19;5:10410. doi: 10.1038/srep10410 (PMC4437377; doi:10.1038/srep10410)
Supplement: Supplementary Information [file srep10410-s1.doc]

**Hydrothermally Grown In-doped ZnO Nanorods on p-GaN Films for Color-tunable Heterojunction Light-emitting-diodes**

Geun Chul Park^1^, Soo Min Hwang^1^, Seung Muk Lee^1^, Jun Hyuk Choi^1^, Keun Man Song^1^, Hyun You Kim^2^, Hyun-Suk Kim^3^, Sung-Jin Eum^4^, Seung-Boo Jung^1^, Jun Hyung Lim^1^ & Jinho Joo^1^

e-mail: jinho@skku.edu & lanosjh@gmail.com

^1^School of Advanced Materials Science and Engineering, Sungkyunkwan University, Suwon, Gyeonggi 440-746, Korea.

^2^Department of Nanomaterials Engineering, Chungnam National University, Daejeon 305-764, Korea.

^3^Department of Materials Science and Engineering, Chungnam National University, Daejeon 305-764, Korea.

^4^HeeSung Material Ltd. 113-9, Yongin, Gyeonggi, 449-884, Korea.


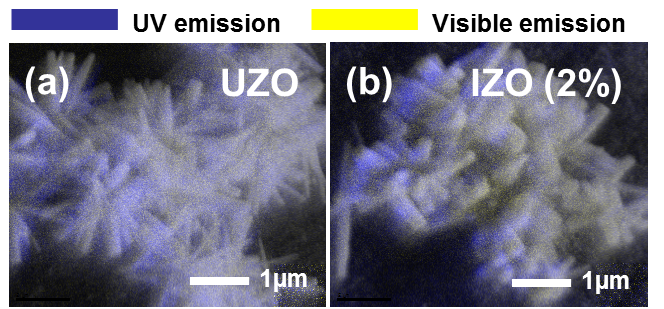


Figure S1. CL images of the (a) UZO and (b) IZO (2%) NRs.


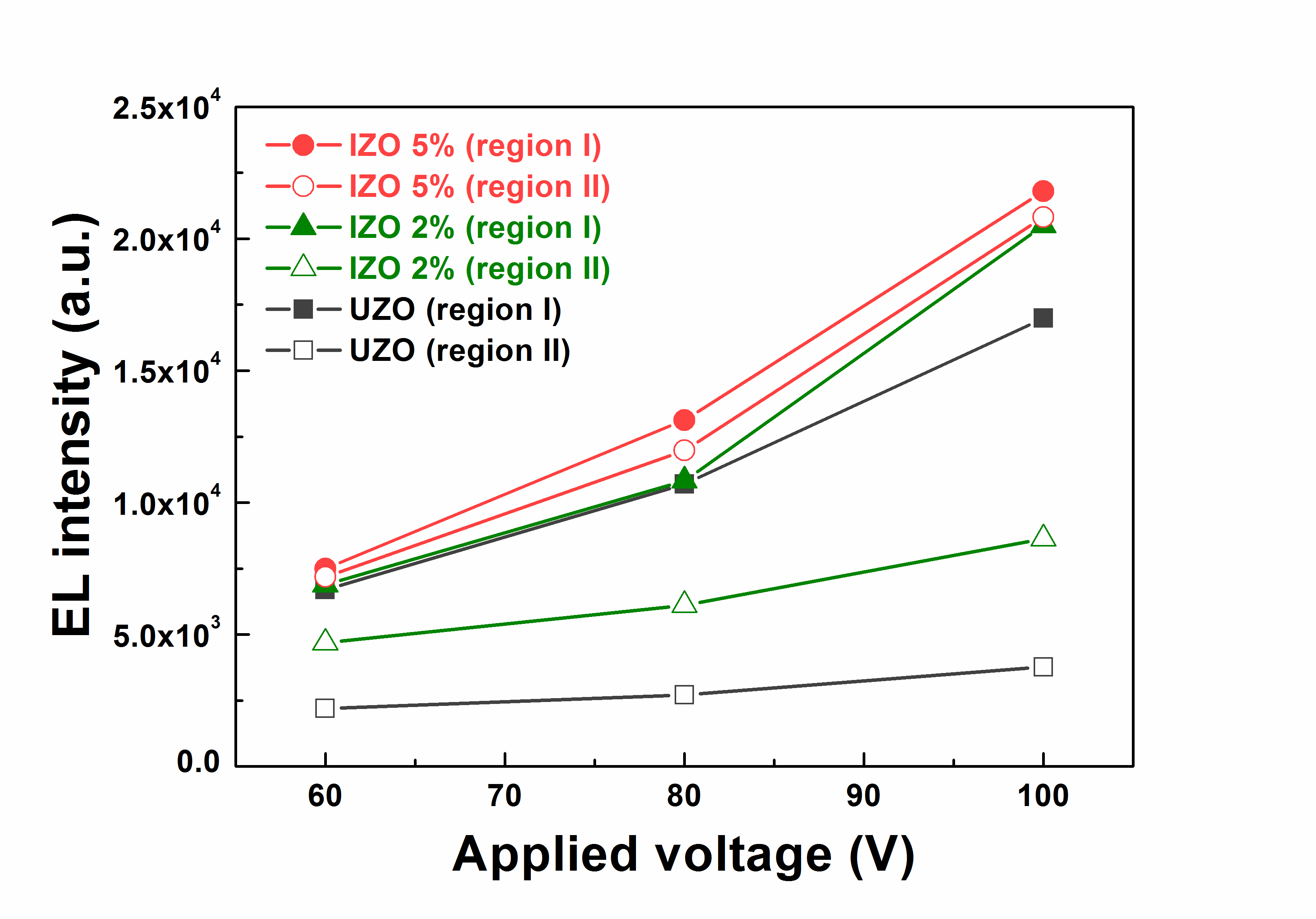


Figure S2. Integrated EL intensity of the UZO and IZO NRs/p-GaN heterojunction LEDs as a function of the applied voltages (60~100 V).


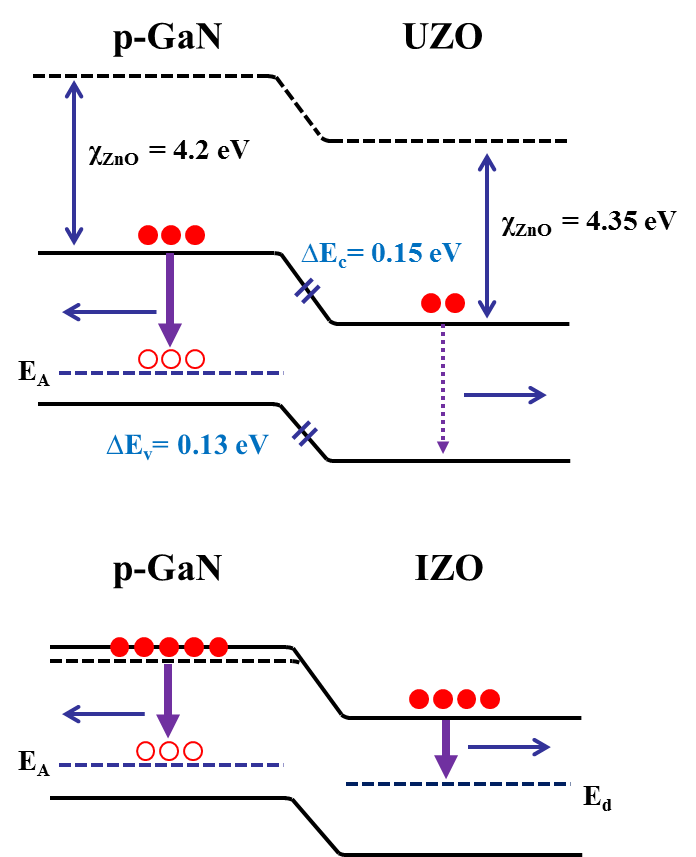


Figure S3. Energy band diagram of the ZnO NRs/p-GaN heterostructure.


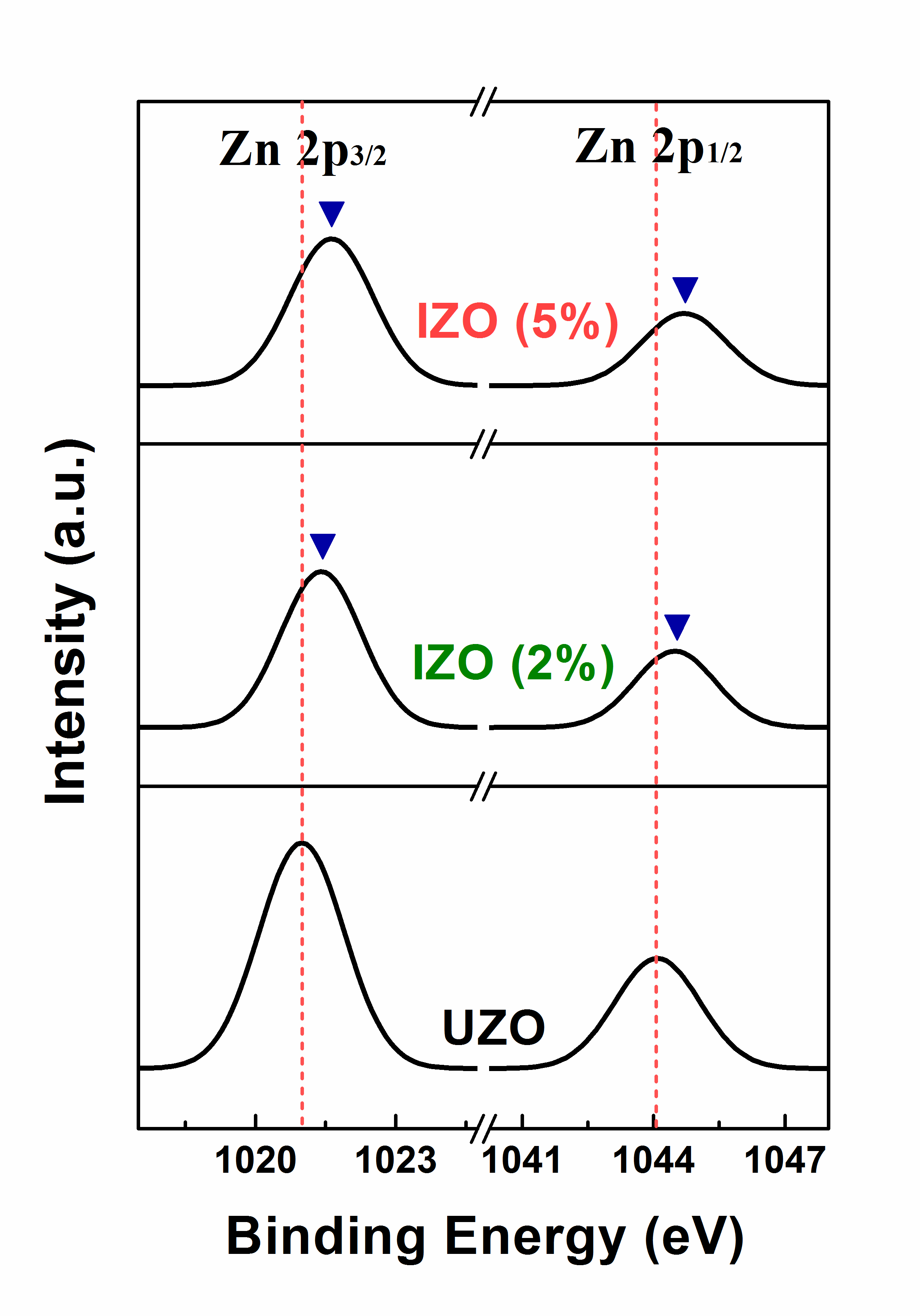


Figure S4. XPS spectra of the Zn 2p peaks of the IZO (0~5%) NRs.
